# Vascular Dysfunction in Malaria: Understanding the Role of the Endothelial Glycocalyx

**DOI:** 10.3389/fcell.2021.751251

**Published:** 2021-11-10

**Authors:** Margaret A. Bush, Nicholas M. Anstey, Tsin W. Yeo, Salvatore M. Florence, Donald L. Granger, Esther D. Mwaikambo, J. Brice Weinberg

**Affiliations:** ^1^Duke University School of Nursing and Durham VA Medical Centers, Durham, NC, United States; ^2^Menzies School of Health Research, Charles Darwin University, Darwin, NT, Australia; ^3^Lee Kong Chian School of Medicine, Nanyang Technological University, Singapore, Singapore; ^4^National Centre for Infectious Diseases, Tan Tock Seng Hospital, Singapore, Singapore; ^5^Hubert Kairuki Memorial University, Dar es Salaam, Tanzania; ^6^School of Medicine, University of Utah and Salt Lake City VA Medical Centers, Salt Lake City, UT, United States; ^7^Duke University School of Medicine and Durham VA Medical Centers, Durham, NC, United States

**Keywords:** glycocalyx, malaria, glycosaminoglycans, endothelium, vascular dysfunction

## Abstract

Malaria caused by *Plasmodium falciparum* results in over 400,000 deaths annually, predominantly affecting African children. In addition, non-falciparum species including vivax and knowlesi cause significant morbidity and mortality. Vascular dysfunction is a key feature in malaria pathogenesis leading to impaired blood perfusion, vascular obstruction, and tissue hypoxia. Contributing factors include adhesion of infected RBC to endothelium, endothelial activation, and reduced nitric oxide formation. Endothelial glycocalyx (eGC) protects the vasculature by maintaining vessel integrity and regulating cellular adhesion and nitric oxide signaling pathways. Breakdown of eGC is known to occur in infectious diseases such as bacterial sepsis and dengue and is associated with adverse outcomes. Emerging studies using biochemical markers and *in vivo* imaging suggest that eGC breakdown occurs during *Plasmodium* infection and is associated with markers of malaria disease severity, endothelial activation, and vascular function. In this review, we describe characteristics of eGC breakdown in malaria and discuss how these relate to vascular dysfunction and adverse outcomes. Further understanding of this process may lead to adjunctive therapy to preserve or restore damaged eGC and reduce microvascular dysfunction and the morbidity/mortality of malaria.

## Introduction

In 2019 there were 229 million cases of malaria and 409,000 deaths despite effective anti-parasitic drug therapy (WHO, 2020). The highest malaria disease burden is in Africa, (94% of cases and deaths), followed by South and South-East Asia (WHO, 2020). Children < 5 years account for ∼2/3 of deaths (WHO, 2020). *Plasmodium falciparum* is the malaria responsible for the vast majority of severe illness and deaths globally (WHO, 2020). *Plasmodium vivax* causes significant morbidity outside of sub-Saharan Africa, and is recognized as causing delayed severe disease and death (Price et al., 2020). Although less common, the simian parasite *Plasmodium knowlesi* causes zoonotic malaria in Southeast Asia, which is frequently severe and sometimes fatal (Grigg et al., 2018).

The malaria life cycle in humans starts with the bite of an infected female *Anopheles* mosquito. Injected sporozoites quickly pass into the liver, multiply asexually within hepatocytes, and develop into merozoites over ∼7 days. After this asymptomatic period, merozoites rupture from the hepatocytes and invade red blood cells (RBC) where they multiply. Rupture of infected RBC (iRBC) causes fever and release of new merozoites which invade other RBC. This erythrocytic/asexual cycle then repeats (Miller et al., 2013).

Interaction between the iRBC and the vascular endothelium results in key features of pathogenesis (Miller et al., 2013). In *P. falciparum* infections, iRBC express binding proteins on the RBC membrane (e.g., PfEMP1) and develop knobs that facilitate adherence to vascular endothelium resulting in parasite sequestration in microvessels (Miller et al., 2013). Vascular dysfunction is associated with endothelial activation, impaired perfusion/oxygenation, as well as organ dysfunction. Endothelial activation, microvascular dysfunction, and reduced availability of the endogenous vasodilator nitric oxide (NO) are related to disease severity and outcome (Anstey et al., 1996; Yeo et al., 2007, 2014, 2017). Microvascular sequestration and endothelial activation are independent predictors of impaired perfusion, severe disease, and death (Hanson et al., 2015), but the precise mechanisms of vascular dysfunction in malaria remain unclear.

The endothelial glycocalyx (eGC) serves protective and homeostatic roles in the vasculature (Villalba et al., 2021). Major structural components of the eGC are glycosaminoglycans (GAG), including chondroitin sulfate (CS), heparan sulfate (HS), and hyaluronic acid (HA), and proteoglycans such as syndecans and glypicans (Weinbaum et al., 2021). Of particular interest in malaria, the eGC can modulate cellular adhesion of platelets and iRBC to endothelial cells (Chappell et al., 2014; Hempel et al., 2017; Introini et al., 2018). In placental malaria, CS and syndecan-1 support cytoadhesion/sequestration after syncytiotrophoblast denudation (Hromatka et al., 2013; Ayres Pereira et al., 2016; Fried and Duffy, 2017). eGC regulates blood flow-mediated NO production by endothelial NO synthase (eNOS) (Florian et al., 2003; Yen et al., 2015; Weinbaum et al., 2021). Shear stress generated by flowing blood can activate eNOS through a mechano-transduction pathway mediated by eGG (specifically HS and the proteoglycan glypican-1). Downstream effects of this pathway include phosphorylation of PECAM-1 and eNOS, which ultimately results in synthesis of NO (Weinbaum et al., 2021).

Breakdown of eGC occurs in certain infectious diseases in which blood borne pathogens (and host factors they induce) come into contact with eGC. Loss of eGC reported in these diseases relates to pathology, disease severity, and adverse outcomes. In sepsis, blood and urine levels of eGC breakdown products are associated with adverse outcomes including organ dysfunction and mortality (Schmidt et al., 2016; Uchimido et al., 2019). In dengue infection, eGC breakdown is related to increased vascular permeability and disease severity (Tang et al., 2017). More recently, injury to eGC has been reported in patients with severe COVID-19 infection (Stahl et al., 2020; Queisser et al., 2021).

**FIGURE 1:**
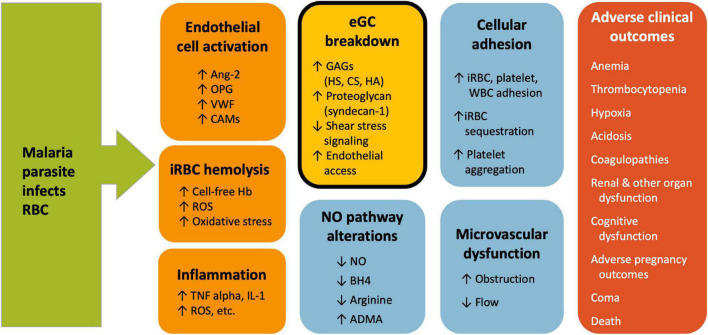
Vascular dysfunction in Malaria. Multiple vascular events contribute to malaria pathogenesis and lead to adverse clinical outcomes. The exact mechanism and sequence of events is not known, but early events include endothelial cell activation and rupture of infected RBC. These lead to endothelial glycocalyx breakdown, reduced NO, cellular adhesion and microvascular dysfunction. Endothelial glycocalyx breakdown (shown in the gold, bolded box) is associated with multiple markers of malaria disease severity and may play a central role in different aspects of malaria pathogenesis. ADMA, asymmetric dimethylarginine; Ang-2, angiopoietin-2; BH4, tetrahydrobiopterin; CAMs, cell adhesion molecules; CS, chondroitin sulfate; eGC, endothelial glycocalyx; GAG, glycosaminoglycan; Hb, hemoglobin; HA, hyaluronic acid; HS, heparan sulfate; iRBC, infected red blood cell; NO, nitric oxide; OPG, osteoprotegerin; ROS, reactive oxygen species; S1P, sphingosine-1-phosphate; VWF, von Willebrand factor.

Given the close overlap of eGC functions with key pathophysiological characteristics of malaria, including cytoadhesion and reduced NO bioavailability, it is logical to suspect potential involvement of the eGC in malaria (Figure 1). The purpose of this review is to summarize evidence for eGC breakdown in malaria and describe how this may relate to pathogenesis of microvascular dysfunction and adverse clinical outcomes. Pre-clinical and clinical studies in malaria are listed in Table 1.

**TABLE 1:** Studies of eGC in malaria.

**Pre-clinical**	**Clinical**
Mouse cerebral malaria (Hempel et al., 2014)	*P. falciparum* in Indonesian adults (Yeo et al., 2019b)
Mouse cerebral malaria (Hempel et al., 2019)	*P. falciparum* in Tanzanian children (Yeo et al., 2019a)
CD36 binding *in vitro* (Hempel et al., 2017)	*P. falciparum* in Tanzanian children (Lyimo et al., 2020)
iRBC cytoadhesion and flow characteristics (Introini et al., 2018)	*P. falciparum* or *P. vivax*, experimentally induced in human volunteers (Woodford et al., 2020)
	*P. vivax* and *P. knowlesi* in Malaysian adults (Barber et al., 2021)
	*P. falciparum* in Tanzanian children (Bush et al., 2021)

## Experimental Animal Studies of the Endothelial Glycocalyx in Malaria

Hempel et al. (2014) first reported loss of eGC in malaria using a mouse model of cerebral malaria (CM). Plasma GAG levels were significantly increased, and transmission electron microscopy showed complete or partial loss of eGC in mice with CM (Hempel et al., 2014). Daily plasma GAG levels suggested that shedding of eGC begins early in the infection (Hempel et al., 2014). Follow-up studies with the murine experimental CM model demonstrated loss of GAG (HA, HS, CS) and proteoglycans (syndecan-1 and 4) in the brain with corresponding increases in plasma concentrations of these eGC constituents, as well as an increase in vascular permeability (Hempel et al., 2019). The authors showed that dexamethasone or antithrombin-3 prevent the eGC loss and certain disease manifestations (Hempel et al., 2019). While the murine cerebral malaria model is commonly used in malaria research, questions have been raised about suitability of the model for translation of results to clinical disease in humans (White et al., 2010; Craig et al., 2012). Challenges with the model include use of different species of parasite and innate differences between mice and humans in disease manifestations and pathophysiology (notably characteristics of adherence of iRBC to endothelial cells and degree of sequestration) (White et al., 2010; Craig et al., 2012). While certain treatments have shown to be effective in the mouse CM model, thus far the findings in mice have not proven helpful in identifying beneficial therapeutics for adjunctive treatment in humans.

## Cytoadhesion Studies

Several *in vitro* studies have demonstrated that cytoadhesion is increased upon loss of eGC. Hempel et al. (2017) studied *in vitro* binding to Chinese hamster ovary cells expressing CD36, a known binding receptor for falciparum-infected RBCs. They found that cytoadhesion was inhibited by presence of eGC, suggesting that loss of eGC may have a role in cellular adhesion process by facilitating the ability to interact with binding receptors such as CD36.

Using a “microfluidic organ-on-chip” model designed to mimic human microvessels, adhesion of iRBC to human umbilical vein endothelial cells (HUVEC) was enhanced following enzymatic degradation of the eGC, compared to adhesion of healthy RBC (Introini et al., 2018). Altered dynamics of the iRBC, including flipping and decreased flow velocity, were also reported, consistent with altered hemodynamics that contribute to cytoadhesion of iRBC to endothelium (Introini et al., 2018). These cytoadhesion studies suggest that the eGC is integral in regulating cytoadhesion of iRBC.

## Clinical Studies in Human Malaria

The eGC is required for induction of NO formation by blood flow over endothelial cells (Florian et al., 2003; Yen et al., 2015; Weinbaum et al., 2021). Our prior studies have documented that host NO production is associated with protection against severe disease in falciparum malaria (Anstey et al., 1996; Yeo et al., 2007, 2014), vivax malaria (Barber et al., 2016), and knowlesi malaria (Barber et al., 2018). Thus, we performed studies to determine if eGC is damaged in humans with malaria from each of *Plasmodium* species known to cause severe disease.

We initially reported evidence of degradation of the eGC in falciparum malaria in 129 Indonesian adults (Yeo et al., 2019b) and 100 Tanzanian children (Yeo et al., 2019a); and subsequently in 146 Tanzanian children (Bush et al., 2021). In both children and adults, there were significant increases in total sulfated GAG in urine. We further characterized the elevated urinary GAG constituents by mass spectrometry and found predominantly HS and CS (but not dermatan sulfate) to be elevated in malaria patients (Yeo et al., 2019a,b), consistent with the composition of GC from the endothelium (Weinbaum et al., 2007). In children, longitudinal analyses showed that urine total GAG levels decreased daily with treatment and normalized by Day 3 (Yeo et al., 2019a). Total GAG were significantly associated with markers of malaria disease severity including parasitemia, parasite biomass, cell-free hemoglobin, and plasma TNF (Yeo et al., 2019a,b). Markers of endothelial activation such as ICAM-1, E-selectin, and Ang-2 were also associated with eGC breakdown products (Yeo et al., 2019b). Breakdown of eGC may expose ICAM-1 and E-selectin, endothelial adhesion molecules implicated in cytoadherence of *P. falciparum*, and exacerbate microvascular sequestration (Hempel et al., 2017; Introini et al., 2018). In both adults and children, measures of NO bioavailability and vascular function were inversely related to the eGC breakdown products (Yeo et al., 2019a,b). In adults, degradation of eGC was associated with mortality (Yeo et al., 2019b). The plasma core protein syndecan-1 was also significantly elevated in adult patients and associated with various markers of disease severity (Yeo et al., 2019b). Degradation of eGC has been implicated in the pathogenesis of acute kidney injury in sepsis (Schmidt et al., 2016). The greater magnitude of eGC degradation in acute kidney injury seen in severe falciparum malaria (Yeo et al., 2019b) supports a role for glomerular eGC degradation in the pathogenesis of acute kidney injury in malaria.

Lyimo et al. (2020) used incident dark field imaging of the microvasculature in the buccal region of the mouth along with biochemical measures including GAG and syndecan-1 to demonstrate eGC loss in African children with malaria. Imaging was used to calculate the perfused boundary region (PBR), a measure used to assess the ability of perfused RBC to penetrate the eGC. The PBR measurement for children with either uncomplicated or severe malaria was significantly increased compared with healthy children, supporting breakdown of the eGC barrier (Lyimo et al., 2020). Additional findings detected on imaging in the malaria patients included impaired vascular integrity, stagnant erythrocytes external to blood vessels, perivascular hemorrhages, and sequestration of late-stage parasites within vessels (Lyimo et al., 2020). Similar to other clinical studies, biochemical markers of eGC loss were elevated in the malaria patients. However in these studies, measures of eGC degradation were slower to recover, with eGC integrity remaining impaired for at least 2 weeks. Biomarkers of endothelial activation in plasma (i.e., angiopoietin-1/2 and thrombomodulin) were also significantly altered in malaria patients (Lyimo et al., 2020).

While most studies to date have focused on breakdown of eGC in falciparum malaria, there is also evidence for breakdown of eGC in vivax and knowlesi malaria (Barber et al., 2021). In Malaysian adults with either vivax or knowlesi infection, levels of eGC breakdown products were significantly increased compared to healthy controls and in knowlesi malaria, were significantly correlated with disease severity (Barber et al., 2021). In knowlesi malaria, there is also significant correlation with biomarkers of endothelial activation [angiopoietin-2 and osteoprotegerin (OPG)] and measures of microvascular function. In the patients with knowlesi malaria, the eGC breakdown marker syndecan-1 was also significantly related to acute kidney injury, supporting the notion that glomerular glycocalyx degradation is a key mechanism of acute kidney injury in malaria and other acute infections.

## Clinical Evidence in Experimentally Induced Human Malaria

The study of healthy volunteers inoculated with malaria parasites presents a unique opportunity to observe events early in infection, compared with clinical trials in which patients have had infection for some time prior to diagnosis. Subjects are inoculated with infected RBC following which parasitemia develops. At onset of clinical signs or ∼7 days after inoculation, the patients receive antimalarial drug treatment. In 45 healthy subjects inoculated with *P. falciparum* or *P. vivax*, biomarkers of endothelial activation, including von Willebrand factor and OPG, increased from baseline to the start of antimalarial treatment, after which levels declined back to baseline (Woodford et al., 2020). In the *P. falciparum* group, Ang-2 increased significantly. However at these earlier timepoints of infection, there were no changes observed in biochemical markers of eGC breakdown, abnormalities using sidestream dark field microscopy of sublingual vessels, or microvascular function as assessed by peripheral arterial tonometry or near-infrared spectroscopy (Woodford et al., 2020). Our study suggests that endothelial activation precedes eGC breakdown and vascular dysfunction, and it also supports potential involvement of Ang-2 as a mediator of eGC breakdown.

## Mechanisms of Endothelial Glycocalyx Degradation in Malaria

While the mechanisms of eGC loss in malaria are not certain, a number of mediators are implicated. There is evidence that angiopoietin-2 contributes to eGC degradation. Elevated plasma levels of Ang-2 have been demonstrated in malaria and are associated with endothelial activation and disease severity (Yeo et al., 2008; Conroy et al., 2009). In falciparum malaria (Yeo et al., 2019b), vivax malaria (Barber et al., 2021), knowlesi malaria (Barber et al., 2021), and other disease settings including sepsis (Beurskens et al., 2020), Ang-2 has been associated with eGC loss, independent of other potential mediators. And in cultured endothelial cells, Ang-2 induces a heparanase-dependent degradation of eGC (Lukasz et al., 2017). Loss of eGC results when the breakdown predominates over new eGC synthesis. Heparanase as well as other substances (e.g., membrane or matrix metalloproteases, thrombin, plasmin, and hyaluronidases) have been implicated as potential “sheddases” that are responsible for eGC breakdown clinically (Becker et al., 2015; Iba and Levy, 2019). Endothelial NOS (eNOS) can prevent the induction of heparanases, with heparanase induced by the eNOS inhibitor asymetric dimethylarginine (ADMA) (Garsen et al., 2016). The eNOS-inhibitors OPG and ADMA are elevated in both falciparum and knowlesi malaria (Yeo et al., 2010; Barber et al., 2021), and both are associated with eGC degradation in malaria (Barber et al., 2021). ADMA and OPG may therefore contribute to induction of heparanase in malaria and to eGC degradation. Oxidative stress is elevated in severe malaria (Rubach et al., 2015; Yeo et al., 2015), and can also mediate glycocalyx breakdown (Pillinger and Kam, 2017). Hemolysis in malaria results in circulating, cell-free plasma hemoglobin that is associated with oxidative stress (Plewes et al., 2017) and eGC breakdown, independent of disease severity (Yeo et al., 2019b). Heme-mediated oxidative stress likely also contributes to glycocalyx breakdown in malaria. The lipid mediator, sphingosine-1-phosphate (S1P), protects against eGC damage by inhibiting syndecan-1 shedding and increasing glycocalyx synthesis (Zeng et al., 2014). S1P is decreased in malaria (Finney et al., 2011; Punsawad and Viriyavejakul, 2017; Yeo et al., 2019b; Barber et al., 2021), and in falciparum malaria is inversely associated with eGC degradation (Yeo et al., 2019b). This suggests that reduced S1P may also contribute toward loss of glycocalyx integrity in malaria. Since erythrocytes are a major source of S1P, malaria-associated anemia could enhance deficiencies in S1P signaling pathways.

Further understanding of which sheddases are involved in malaria could identify potential pharmacologic targets leading to new adjunctive treatments. Better understanding of the mechanisms will also help determine how eGC loss affects factors related to both eGC function and malaria pathogenesis (including NO formation in response to shear stress of flowing blood and the processes of cellular adhesion to endothelium by leukocytes, platelets and iRBC).

An interesting question is whether the eGC breakdown products could themselves have detrimental effects and contribute to pathogenesis, rather than being simply being inert, circulating GAGs and proteoglycans. This concept has been reported in sepsis in which GAG are linked to cognitive sequelae. In a mouse model, circulating GAG fragments were shown to cross the blood brain barrier, bind to brain-derived neurotrophic factor (BDNF), and inhibit BDNF-mediated long-term potentiation in the hippocampus, a mechanism underlying memory (Hippensteel et al., 2019). Particular sulfation patterns of HS were associated with better binding to BDNF (Hippensteel et al., 2019). Interestingly, in patients with sepsis in the intensive care unit, these same sulfation patterns in plasma HS predicted cognitive impairment lasting up to 2 weeks after discharge (Hippensteel et al., 2019). Lyimo et al. showed that in children with cerebral malaria, plasma concentrations of HS were significantly higher in those with deeper coma (Lyimo et al., 2020). It is possible that eGC degradation not only exacerbates parasite sequestration and microvascular dysfunction in cerebral malaria, but through elevated circulating HS, may compound coma severity in cerebral malaria. Another example of pathological changes related to breakdown products themselves is in COVID-19 in which endothelial barrier dysfunction can be induced by HA fragments (Queisser et al., 2021).

A therapeutic that could prevent loss or promote formation of eGC could potentially be used as adjunctive therapy in patients with malaria. There are a number of proposed therapeutics to ameliorate eGC loss by mechanisms such as preventing shedding, inhibiting putative sheddases, or regenerating eGC (e.g., albumin, sulodexide, sevoflurane, rhamnan sulfate) (Iba and Levy, 2019; Weinbaum et al., 2021). Data regarding these agents are mainly based on experimental animal studies, and there is no clinical evidence to support their clinical use currently. Of relevance to malaria, sevuparin is a modified low molecular weight heparin-like molecule without anticoagulant properties and with close similarity to the glycosaminoglycan HS. Sevuparin and other heparin analogs inhibit rosetting (binding of iRBCs and RBCs) and cytoadhesion of iRBC to endothelium *in vitro* (Vogt et al., 2006; Leitgeb et al., 2011; Saiwaew et al., 2017). In a Phase I/II clinical trial in patients with uncomplicated malaria, sevuparin inhibited parasite invasion of RBC and reversed sequestration of iRBC (Leitgeb et al., 2017).

## Conclusion

There is growing evidence that loss of eGC is associated with vascular dysfunction and disease severity in malaria. This loss may lead to multiple vascular events and adverse clinical outcomes in malaria (Hempel et al., 2016; Georgiadou and Cunnington, 2019). Many questions remain including the need for further understanding of the mechanisms underlying eGC breakdown in malaria. We need to identify pathways responsible for eGC breakdown, e.g., the initial steps and role of the parasite or host factors as triggers; whether the breakdown products themselves are detrimental contributors to pathology; and the relation and kinetics of eGC synthesis vs. breakdown in malaria. Clinically, it will be important to determine if rapid assays of eGC breakdown products (e.g., urine total GAG) can provide helpful prognostic and treatment insights, and whether adjunctive agents that prevent loss or replace eGC can ameliorate or prevent severe disease.

## Author Contributions

All authors listed have made a substantial, direct and intellectual contribution to the work, and approved it for publication.

## Conflict of Interest

The authors declare that the research was conducted in the absence of any commercial or financial relationships that could be construed as a potential conflict of interest.

## Publisher’s Note

All claims expressed in this article are solely those of the authors and do not necessarily represent those of their affiliated organizations, or those of the publisher, the editors and the reviewers. Any product that may be evaluated in this article, or claim that may be made by its manufacturer, is not guaranteed or endorsed by the publisher.

## Abbreviations

ADMA: asymmetric dimethylarginine
Ang-2: angiopoietin-2
BDNF: brain-derived neurotrophic factor
CM: cerebral malaria
CS: chondroitin sulfate
eGC: endothelial glycocalyx
eNOS: endothelial nitric oxide synthase
GAG: glycosaminoglycan
HS: heparan sulfate
HA: hyaluronic acid
iRBC: infected red blood cell
NO: nitric oxide
OPG: osteoprotegerin
PfEMP1: *P*. *falciparum* erythrocyte membrane protein 1
RBC: red blood cell
S1P: sphingosine-1-phosphate.
